# The Thirty-First Annual Meeting of the American Dental Association

**Published:** 1891-11

**Authors:** N. S. Hoff

**Affiliations:** Ann Arbor, Mich.


### THE DENTAL REGISTER.

VOL. XLV.] NOVEMBER, 1891. [No. 11.

### COMMUNICATIONS.

### The Thirty-First Annual Meeting of the American Dental Association.

SARATOGA SPRINGS, N. Y., AUGUST 4, 1891. DENTITION OF THE FELIDÆ, BY A. H. THOMPSON, TOPEKA, KA.

Reported by N. S. Hoff, D.D.S., Ann Arbor, Mich.

*Continued from page* 446.

The dentition of the cat is interesting because of its high specialization. The extremes of form in the teeth of animals indicate the possible variation in less highly specialized teeth where compromised forms obtain because of more variety in diet. The kind of food determines the degree of specialization. Hence we have in carnivora and herbivora examples of extremes in specialization. The general characteristics of the carnivora are small incisors, large canines with long roots, wide diastema, reduction of the molar series, cutting blades on the molars, etc. In no other group.of animals are the jaws so well and variously armed with teeth. The jaws are short, muscles strong, and the movement limited to the verticle. Food is not masticated or insalivated; being raw flesh it is only necessary that is should be torn and swallowed as it is easily digested.

The most highly specialized teeth in the felidæ are the sectional premolars and the prehensile canines, which are so characteristic as to place this family at the head of the carnivora in specialization.

The paper gives at considerable length the peculiar and important characteristics of the felidæ teeth, making an exceedingly interesting study in comparative odontology.

DISCUSSION.

Dr. W. C. Barrett wished to congratulate the association on the fact that its members were turning their attention to scientific subjects in which they ought to be especially interested, and hoped this paper would receive a careful consideration. Dr. Barrett thinks the true archetype is to be found in the dentition of the ungulata, as seen in the pig, where the dentition is represented by 48 teeth. Of the carnivora the dog tribe have a more highly specialized dentition than the felidæ, or cat tribe. He does not think the kind of food a factor in the specialization of this dentition.

Dr. C. N. Peirce: We may draw practical conclusions from such papers that are of interest and value as well. There is undoubtedly a general resemblance in the dentition of animals living upon similar food, and it has been noticed that where the dentition has been specialized in form because of diet, that there is a decided tendency toward reduction in the number of teeth. In the horse 44 teeth constitute the typical dentition; but in the domesticated and blooded horse, the dentition is reduced by 4, possibly due to disease, nervous affections, or food. In the omniverous man his dentition seems to be in a transition state; the tendency of the teeth to decay may be due to poor tooth tissue resulting from this uncertain conditon. There is a notable tendency in the teeth of the Chinese to dwarf the cuspids; this may be due to the common diet of rice, etc. In these people there is also a tendency toward enlargement of the incisors.

It is not likely that the human family will eventually become edentulous, but the habits of the people and the diet will produce modifications in the number as well as in the forms of the teeth, adapting them to the uses required. The character of the food, its consistency, etc., will also have much to do with the character of the tissues developed in the tooth.

Dr. Harrow: In man there is no canine tooth, but the cuspid tooth takes its place, and its function is not to tear food, but it serves as a cutting tooth and as a guide in closing the teeth, thus assisting in securing a perfect closure or articulation.

Dr. Barrett: It is a curious fact that all animals native of

Australia have the marsupial dentition entire, or in marked modification, and imported animals with different dentitions are reduced in size.

Dr. Truman asked Dr Peirce why he thought the third molar was destined to be lost in man's dentition, and why he considered the cuspid to be the canine tooth, changed in form and size?

Dr. Peirce: I have personal knowledge of over twenty patients in my practice who are of sufficient age, but have never erupted the third molar, and know no reason why this ratio should not prevail in every practice. Examinations of gorilla and Hottentot skulls shows plenty of space behind the third molars in every case, and the third molar tooth is large and well developed; while in man the third molar is small, or inconstant and occupies a very limited space.

There is no excessive development of the canine in animals. It is an ancestral development, which time and limited change of circumstances and conditions have not sufficed to cause its modification.

Dr. Brophy: The third molar is not invariably smaller than the other molars; frequently it is larger. I am inclined to think that the lateral incisor is more often lost than the third molar.

Dr. Peirce: The defficiency of the lateral incisor is more likely to be transmitted from parents than the third molar.

Dr. Morgan: If the superior third molar is lost because of non-use, why should we find in the same mouth the superior third molars absent and the inferior third molars present? or why should the lower lateral incisor be present when the superior lateral incisor is gone?

Dr. Rhein: Is it not possible to account for the absence of the superior lateral incisor because of loss of its germ by ancestral harelip?

Dr. J. D. Patterson read a paper entitled,

### DISEASES OF THE ORAL MUCOUS MEMBRANE—ITS PATHOLOGY AND INFLUENCE UPON THE TEETH.

Incipient causes of disease in this membrane may be accidental, constitutional or hereditary. The accidental cause usually obtains while the others predispose and assist. Accidental causes are numerous; mechanical alone or accompanied by infectious material. Disease causing low functional activity are an important factor in all causes. Thermal changes are capable of modifying normal function to the extent of producing disease in the oral mucous membrane by first checking the secretion of the mucous glands, causing determination of blood to the part, effusion of serum, abnormal exfoliation of cells, purulent mucus discharge, and a general breaking down of the structures into pus, then the sub-epithelial tissues become involved and extensive inflammatory condition results. The above stages accompany disease in which constitutional, hereditary and zymotic influences do not necessarily assist, although micro-organisms may be present in the later stages.

"Mouth breathing" is a source of irritation and disease because of thermal changes and evaporation of the moisture necessary to the normal function of the membrane. Uncleanness and the accumulation of calcareous deposits are the most frequent causes of local disturbances.

A catarrhal condition of the mucous membrane of the nasal and oral cavity, because of the continuity of tissue, is likely to produce points of irritation and disease of the gums. Owing to the peculiar structure of the gum margins they are particularly liable to disease by mechanical irritants and infection when there is a catarrhal condition present, producing often the condition called pyorrhœa alveolaris. The catarrhal affection is not a disease, but an impairment of function favorable to the production of disease by other causes. There is no catarrhal diathesis and there is no proportionate relation between a catarrhal condition and pyorrhœa alveolaris. Climate may have a direct influence upon catarrhal affections, but it is not known to have any upon pyorrhœa alveolaris. Catching cold or any thing that will interfere with normal function is capable of producing disease of the mucous membrane of the oral cavity, independent of hereditary conditions, constitutional diathesis, or the influence of mico-organisms.

The irritated mucous membrane, which we find weeping out an abnormal quantity of mucus or muco-pus, is not pathognomic of any special irritant, but simply a condition. Pyorrhœa alveolaris is only a designation of a certain stage in catarrh of the oral mucous membrane, and is not necessarily pathognomic, and may result from a great variety of causes.

Dr. H. A. Smith read a paper entitled,

PHAGOCYTOSIS.

The paper gave a brief statement of the amœboid character of the white blood corpuscle, its history and the reason why the name phagocyte had been given to it. It is by some now thought to be the agency whereby parisitic influences in the tissues of the body are destroyed or overcome. It is probable that these leucocytes are the agents which prevent excssive irritation from septic influences in the practice of filling root canals, especially where the practice of "immediate root filling" is practiced. The reason why bacteria are not found in the living pulp is because of these phagocytes circulating in the blood and permeating the tissues and coming into contact with them, destroy them. In the dead pulp there are no phagocytes and consequently the bacteria proliferate unhindered. Teeth in which the pulp is destroyed by a blow which does not fracture the enamel or dentine but destroys the pulp by the shock, oftentimes remain for years without the pulp, dead though it is, undergoing putrefactive change, or causing inflammatory conditions. This may be due to the fact that the leucocytes were extravasated in such numbers at the time of the accident as to overcome all bacterial influences. The successful treatment of pyorrhœa alveolaris by surgical means may be due to the presence of large numbers of the leucocytes in the consequent highly inflamed tissues. It is hoped that a wider study of this aspect of the germ theory for accounting for all diseases and curative agencies may be made.

DISCUSSION.

Dr. Barrett: It may be true that the phagocyte is the agent which counteracts the influence of the bacteria, by destroying the germ itself, by enveloping it in its peculiar digestive apparatus, where it breaks down its cell wall and appropriates its protoplasm, rejecting the cell wall. This has not been positively demonstrated, but it is probably true.

Dr. Cravens: Is pleased to present his compliments to the phagocyte, as it gives him a scientific reason for his practice of immediate root filling, and accounts for the success he has had with this method of practice.

Dr. Waters: We should not forget that there is always a first cause which must not be overlooked. This first cause should receive first attention and the attending causes should not receive the entire attention, important though they may be.

Dr. Leroy asks if pyorrhœa alveolaris ever effects devitalized teeth. He had seen it stated that it did not.

Dr. Rhein said the worst case he ever saw involved devitalized teeth as much as those with live pulps.

Dr. Crawford does not think that pyorrhœa affects teeth that have had their pulps destroyed, removed, the canals treated and filled. Alveolar abscess is frequently mistaken for pyorrhœa.

Dr. Patterson: Constitutional diathesis is a very important factor in the production of pyorrhœa, but I did not endeavor to discuss it in my paper.

Dr. T. W. Brophy, chairman of Section VII, reported four papers, the first one read was by Dr. W. C. Barrett, entitled,

### A PLEA FOR CONSERVATISM.

The errors into which an unrestrained enthusiasm has led Profs. Koch, Leister and Pasteur, have recoiled and we suddenly discover that all lines of research, except along the line of bacteriology, cannot be ignored in our search after scientific truth. Some years ago on the floor of this society the work of Dr. Miller was discredited or hooted at. But now the truth has more than made its way; in fact the pendulum has swung back too far, and there is danger that we may have an antiseptic craze. The study of the micro-organisms of the human mouth is essential to an intelligent conception of oral pathology and dental medication. But germicides and antiseptics are not the only remedies to be made use of in the treatment of oral diseases. We are apt to run riot when a new idea is discovered.

So much attention has been given to antiseptic agents that curative agents seem to have been laid aside, and when failure to cure results the agent used is blamed and pronounced worthless, when the fault lies with the operator, because he has not recognized the conditions and made the proper selection of a remedy. The immediate root filling fad is an example of how a little knowledge can work a great mischief.

We must realize that in almost no case, which the dentist is called upon to treat, is absolute asepticism possible or practicable. In capping pulps and treating abscesses, antiseptics are useful, but they are not sufficient. Because we cannot successfully exterminate micro-organic influences entirely is no reason why antiseptics are inefficient; they are valuable helps toward cures, and must always be used. No practitioner should be content until he possesses the most approved means for sterilizing all instruments used in treatment so as to prevent the possibility of inoculating susceptible tissue. In healthy tissue it may be possible that the phagocytes are capable of destroying all infectious matter that would ordinarily be introduced, but debilitated tissue would become inoculated by the smallest possible amount of infectious material, because there is a scarcity of the phagocytes.

Again it is not enough to deluge a cavity or root canal with detergents, saturate it with germicides, soak it with antiseptics, or even exclude infected salvia by means of rubber-dam. A skillful operation must be made and anything necessary to the accomplishment of this end must not be overlooked. The perfectness of the operation will increase the permanency of the antiseptic treatment. Let us honor and encourage the patient and industrious investigators, who are giving us scientific facts, but condemn the tyro, who catches only a portion of the truth and brings into disrepute the most important scientific truths because of his ignorance and bungling. Earnestly seek new things, prove all, and retain the true and good.

WEDNESDAY EVENING.

At the opening of the session, Dr. Crouse for the Dental Protective Association, made a statement as to the condition of the patent litigation. The next suit to be tried will be the one involving what is known as the Low Bridge patents, any one possessing evidence will oblige the association by communicating at once with Dr. Crouse. The committee appointed last year to examine the books and accounts of the association, reported the affairs of the association well managed and a comfortable balance of $20,000 in good securities, etc.

In lieu of a paper, Dr. Peirce verbally stated a case of a patient of a neighbor dentist having swallowed a large sized rubber-dam clamp, which a surgeon who was called in consultation could not recover. The patient was sent home and instructed to eat bountifully of mashed potatoes exclusively for several days. The result was that the clamp passed through the alimentary canal without doing any harm and was recovered on the second day incysted in a hard ball.

Dr. J. S. Marshall read a paper in which he gave the history of the use of Pyoktanin in the treatment of a case of malignant neoplasm, originating in the antrum, and involving the superior maxillary and malar bones. Two operations had failed to eradicate the difficulty. Parenchymatous injections of pyoktannin were used; strength one part in five hundred of a 0.6 per cent. solution of chloride of soda in distilled water. Twenty minims were injected at first and the amount gradually increased to one drachm. In six weeks the patient was considered well enough to resume his business occupation, and it is confidently thought he is cured. Special antiseptic precautions were observed to prevent inoculation and the formation of sores. In all the treatment, the exhibition of the drug produced no symptoms of systemic derangement.

Dr. J. D. Patterson described a case illustrating some new features in the treatment of a fracture of the inferior maxilla, we are unable to intelligibly describe without reference to the drawing accompanying the case.

Dr. Truman W. Brophy described his method of closing the fissure in cleft palate. Always operate when the patient is young in order to succeed. Described a case where he had operated on a babe three weeks of age. Two holes were first drilled through the superior maxillary bones from the buccal surface on one side, passing through the bottom of the nasal cavity just above the palatal plate to the opposite buccal surface; one of these holes was toward the front and the second farther back and under the malar bone. The soft palate was closed with sutures in the usual way, and the edges of the palatal plate pared; a wire was then passed through one of the holes that had been drilled and back through the other; the two ends were then brought together and twisted until the edges of the bone had been brought into contact and the wires left until union had taken place. The bones at this age yield readily and when brought together by the wire compress they are held in place definitely until nature has had time to unite them permanently.

DISCUSSION.

DR. MARSHALL thought such an operation on a child three weeks old was too severe, and he should fear a fatal result from shock. The germs of the teeth would be liable to destruction from the drilling and manipulation of the wire.

DR. CRAVENS did not think the development of the teeth would be interfered with as they were already too far advanced.

DR. VAUK has had two fatal experiences from operations on children for cleft palate, and he would be inclined to say that such an operation was extra hazardous.

DR. MARSHALL related a case in which he had operated successfully for cleft of the soft palate on a patient forty-nine years of age.

DR. BROPHY said that children were less liable to suffer from shock than adult persons, and the soft or semi-gelatinous condition of the bones of the face at this time of life made it possible to bend them and close the cleft with certainty. It is quite important in these operations that the tissues be brought into definite and close contact, and to be sure to get this it may be advisable to lap them sufficiently to prevent separation by any possible movement.

DR. VAUK said that it was not desirable to cut entirely through the soft palate in drawing it together, but make slits which will permit stretching.

Under Section I., Dr. D. R. Stubblefield read a paper on

PEROXIDE OF HYDROGEN.

The Doctor had made careful analyses of several prominent manufacturer's products, with the view of determining the agents which are the active cause in corroding or dissolving hard tooth tissues. In three of the most prominent preparations he found both hydrochloric and sulphuric acid, and none of them contained any ozone. One preparation when evaporated left a white residue which charred when strongly heated. If the profession is restricted to the use of such deceptive preparations, over reliable signatures stating that they contain no acids, how can we expect anything but disastrous results from the use of this drug?

Referring to the case reported at the meeting last year by Dr. Peirce, where a large amount of exostosis on the root of a tooth had been completely dissolved away and the cementum and enamel attacked, after being in a bottle of peroxide of hydrogen for three weeks; the peroxide was renewed with fresh every other day, Dr. Stubblefield said this was due to the sulphuric acid, as there was an insoluble white precipitate, the chloride of calcium would be readily dissolved in the fluid and there would be no precipitate. Dr. Stubblefield does not think there is a neutral preparation of peroxide on the market. The ebullition of gas is observed in root canals containing no pus or blood, and in experiments made with blood and pus outside of the mouth, it was found that a degree of heat corresponding to body heat was necessary to secure ebullition; indicating that the presence of blood and pus were not necessary to produce decomposition of the hydrogen, but that warmth was a factor. Again neutral peroxide of hydrogen was injected into root canals containing blood and pus and no ebullition was observed; and hydrochloric acid alone was used in root canals containing blood and pus, and the same kind of ebullition was observed as when acid peroxide of hydrogen was used. It would seem that the acid peroxide has a valuable place because of the acid it contains as a detergent and disinfectant, but it would seem a wise precaution to follow its use with an anti-acid or neutralizing preparation when used on the teeth.

DISCUSSION.

Dr. Peirce: The tooth I exposed to the action of the peroxide of hydrogen was largely exostosed. I was very much surprised to find the exostosis all removed, but the enamel and dentine scarcely affected at all.

Dr. F. Abbott: I was much pleased with Dr. Stubblefield's paper, but it makes me sad to think that I have been relying upon peroxide of hydrogen as a sure test for the presence of pus in deep-seated and obscure places.

Dr. Vauk: It seems to me it is an easy matter to test the different preparations with litmus, and when an acid preparation is found, use it with caution or take precautions to prevent its doing harm. It is a very unstable preparation.

Dr. Friederichs: The very fact that the heat of the mouth is sufficient to decompose this material makes it the valuable preparation it has proven to be; by its decomposition gases are evolved which force their way to the surface, carrying with them foreign and dead material which we want to get rid of.

Dr. Truman: Notwithstanding this adverse report we must not give up the use of peroxide, for the readiness with which it decomposes and gives up its elements makes it invaluable not only as a detergent, but as a germicide and disinfectant through the liberated oxygen, sulphuric acid, etc.

Dr. Ingersol: This preparation is not a chemical compound; the extra oxygen is present because of force used in the preparation, and consequently it is easily liberated when even slightly warmed. Peroxide of hydrogen is not a medicament at all because it possesses no stimulating property whatever. It behaves very badly in closed cavities, antrum, and blind abscesses, because it coagulates the pus in the cavities, and this coagulated mass becomes an intolerable irritant. Don't like it in pyorrhœa or any case where there is pus.

Dr. Horton suggested using a dilute solution in warm water to wash out the pus before using the peroxide in full strength.

Dr. Stubblefield: Peroxide of hydrogen as at present prepared has no special advantage over aromatic sulphuric acid, which will accomplish all that peroxide will do and has a stimulating or medicinal virtue besides. The pain accompanying its use is due to the combination of the water and sulphuric acid, producing heat.

Dr. E. Parmly Brown described a new method of making a porcelain crown with a collar around the end of root A collar is made of platinum to fit the end of the root, and the band is left wide enough so that it may be cut in slits all around, down to the face of the previously prepared root; the projections are then bent down, one at a time, over the end of the root forming a complete covering or cap. A Brown or Logan crown is then placed in position, the pin passing through the cap with the pulp canal, and cap and tooth crown removed together and fused together with porcelain in the furnace. The cap may also be soldered on to the crown.

Dr. Louis Ottofy made the report of Section II, on Dental Education, Literature and Nomenclature.

At the present time there are in existence thirty-three dental colleges; 1,241 students graduated from these colleges this year, being an increase of two hundred and seventy. Twice as many students are graduated now annually as five years ago. Thirteen colleges graduated this year less than 100 students; eight graduated 250, and twelve graduated 900. There are 103 dental societies in the United States, distributed as follows: 4 national, 7 inter-State, 41 State, 22 district and local and 29 city. The total enrollment of all these societies is 4,000. Eighty-two of these societies were unrepresented in the American Dental Association last year.

The Post-Graduate Dental Association, organized on the Chautauqua plan, is a promising institution; its course of study and reading embraces a special and definitely outlined course of reading in dental literature, which when completed will entitle the reader to an examination, which if satisfactory will secure a certificate.

Dr. Peirce read a paper written by Dr. Charles E. Koch, of Chicago, on

THE STATE BOARDS, THE PEOPLE'S OFFICERS AND THE PROFESSION.

All agree that dentists should be better educated. National law provides that the several States of the Union shall each make its own laws for the regulation of the conduct of its citizens. Accordingly each State has enacted its own law regulating the practice of dentistry. People do not judge a profession by its brightest lights, but by its members in the aggregate. Therefore the people, for self-protection, demand that the standard of dental education shall be sufficiently high to indicate that the possessor of its diploma is qualified and trustworthy. As educators we ought to recognize the fact that the future dentist is to serve the people intelligently and faithfully, and no student should be encouraged to enter the profession who has not the necessary qualification of body and mind and soul to serve the people faithfully in this particular sphere. If this idea was enforced when a student entered college and all through his course he was closely scrutinized, and not graduated until qualified, the diploma of a dental college would have some significance. While the States do not supervise the work done in the colleges some of them review it by requiring graduates to pass an examination before their State boards before allowing them to practice. This is an additional safe-guard for the people, and it is equitable to all concerned. It protects the college which is doing honest work and compels the mere money-making organizations to take better ground than they otherwise would. State boards also exercise an important function in protecting the people from charlatans and tyros. The charge has been made that these boards are generally appointed through political influence and are liable to be incompetent if not corrupt. The writer has never known of an obscure, unintelligent or dishonorable man being appointed to one of these boards. Just discriminations are made between the work done by the large number of competent teachers and that done in schools run by unqualified pretenders on the money basis only. This the profession and the public expect from State boards. The number of dental schools has unreasonably increased during the past few years, and since the standard in some is so low, the people have been compelled in order to protect themselves to establish, at least, minimum requirements. Many think some States have gone too far and require too much. But the fact is in most States the requirement falls short of what it should be. The profession instead of finding fault with the work of these boards should support and sustain them. Recruiting the future dental profession must be done with more circumspection than ever before, and if only qualified men are allowed to enlist, it will be impossible for politicians to appoint unqualified men on examining boards. The mere possession of a diploma, although from a regularly chartered institution, brings no vested right. The people have the right to demand that the professional man who is licensed to practice shall be fully qualified. The writer thinks every State should do so, if for no other reason that colleges which are doing disreputable work may be exterminated.

It is charged that State examining boards assume greater virtue and knowledge than college faculties. This is not true. State examining boards do not attempt to impart instruction, and they in comparison do only what the county examining boards do for candidates for teachers in the public schools; in this case graduates as well as non-graduates, even of schools maintained at public expense entirely, are subjected to the same examination. If it is necessary to examine the graduates of publicly maintained institutions, how much more is it necessary with graduates of schools maintained and supported by student's fees. The dental profession must support and sustain its State examining boards if it cares to be considered a respectable profession and have the confidence of the people.

DISCUSSION.

Dr. Crawford: Outside of purely scientific questions dental legislation is the most important one discussed to-day. In regard to the statement that the people are demanding protection from dental charlatans I don't think the people, en masse, are especially interested. The fact is there are not, to-day, enough duly qualified dentists to serve the people as they ought to be. As to the standing of the profession, no profession is more respected, and its schools will take as high, if not better rank, than the medical schools. No State should pass a law compelling its examining board to examine the graduates of every school, but the board should be entitled to register the graduates of schools maintaining a proper standard of qualification. The legislature of Tennessee, to a man, when interviewed, expressed the opinion that all reputable colleges should be recognized.

Dr. Barrett: Examining boards do not know what methods teachers have adopted in giving instruction and it would not be a difficult matter to puzzle and annoy a very bright student, and more so a timid or bashful man, although well qualified. State examiners who have never been teachers know little of the difficulties of imparting instruction, and it may be that members of college faculties could embarrass, without much difficulty, the State examiners themselves. How would it do to have a national board of examiners to examine State boards and college faculties? The college faculties and State boards must be harmonized. Members of State boards should examine the workings of the various colleges, and approve or discredit their work, and then accept or reject their diplomas.

Dr. N. S. Hoff read the report of Section III on Operative Dentistry. The report reviewed the work of the profession during the past year as expressed in the periodical literature, contributions and proceedings of societies, making from these such inferences as were possible as to the general progress in the manipulative branch of practice.

Dr. A. W. McCandless made a similar report on new instruments and appliances, making mention of and exhibiting at the meeting every new appliance or instrument which had been brought to the notice of the profession during the year.

Dr. L. E. Custer read a report on dental pain obtundents and local anæsthetics, and exhibited new appliances for the accomplishment of anæsthesia.

The use of sulphuric ether marks the beginning of the present idea of using volatile agents as obtundents. Dr. Ottolengui suggested and used this agent in 1888. It is applied by means of a spray apparatus which will throw an attenuated spray of ether upon the dry dentine, where by rapid evaporation reduction of temperature is secured, resulting in interruption of normal function; continued use would result in death of the pulp. Nitrous oxide used in the same way is used to obtund sensitive dentine. A difficulty in its use is that a very strong tube is necessary to convey it from the cylinder to the spray apparatus. It is not likely that this agent will become popular because of the necessarily unwieldy apparatus required and the possibilities of damaging results.

Dr. Rhein suggests and has used chloride of methyl for producing low temperatures and obtunding sensitive dentine. It is possible to lower the temperature to 40° below zero with this agent. It has no affinity for water and acts only by lowering temperature. The apparatus for its use is comparatively simple and readily managed. It produces more cold than ether, the pain following its application is briefer and the impression more profound. If inhaled it produces general anæsthesia. It is rather expensive, and smells badly, but it is a valuable agent.

Chloride of ethyl comes from France and is put up in three gram glass flasks or tubes, having an attenuated end, which is broken off when wanted for use and the liquid becomes gaseous and escapes. If this is directed into the sensitive cavity of the tooth it produces insensibility by the rapid evaporation of the fluid. The principal advantage of this agent is the fact that no costly or bulky apparatus is needed for using it.

Another class of agents are desiccating obtundents. Hot air injectors, instruments for spraying the cavity with hot air containing some volatile agent. Of this class of agents only such as use a spray material which has a strong affinity for water are practical or useful, as any anæsthetic property a drug might have ordinarily, is lost in attenuation when used as a spray. A convient apparatus of this character is the "Small Obtunder." It consists of a small cylinder with a heating bulb and proper points. In the cylinder is a cartridge containing absorbent material, saturated with absolute alcohol. The bulb is heated and the vaporized alcohol is injected from the nozzle into the cavity where it acts by the heat and its affinity for water as a desiccant, and producing an obtundent effect by interfering with the normal function of the nerve fibril. The Milton and Richmond devices are constructed on the same principle and are adapted to the use of various agents, essential oils, etc., but as these agents have no special affinity for water, they do not produce desiccation and are inefficient.

*Local anaesthetics.*—New anæsthetics are not as numerous as obtundents. Many nostrums have been put upon the market. Cocaine seems to be the basis of most of them. It is combined with such agents as chloroform, ether, alcohol, carbolic acid, antipyrine, aconite, chloral, etc., etc. Cocaine is a powerful and dangerous drug and is used with the greatest caution, and two per cent. solutions are now generally used when it is injected. Recent examinations of the formulæ of some of these nostrums reveals the fact that many of them are positively dangerous to be used in any quantity and should never be injected into the circulation.

*Refrigeration* by chloride of methyl, nitrous oxide and chloride of ethyl, is being used as a local anæsthetic with gratifying results. The principle of its action is to paralize the neutral activity of the nerve supplying a limited part only. This method of necessity cannot be as effective as injections of cocaine, because of the rapid restoration of normal function by means of the circulation.

Dr. V. H. Jackson, of New York, read a paper on

### A METHOD OF REGULATING TEETH.

Every tooth should be encouraged to take a correct position in the arch when erupting. At this time because of the small amount of alveolar process developed, the teeth are readily moved if out of position. In the experience of the author the wire crib and springs accomplish this most readily. Impressions are best taken in modeling compound, the hardening hastened by applying small napkins wet in ice water. Use pulverized soap-stone to prevent dragging when the teeth are long. Fill all irregular spaces and undercuts with wax previous to taking the impression. If a tooth is inclined so much as to prevent easy withdrawal of the impression, with a string tie soft compound around the tooth so as to secure such an incline as will allow of easy withdrawal of the impression. Before taking the impression lay over this compound two or three pieces of wet, thin paper to keep the warm compound from adhering to the tooth and its bushing of compound. After removing the impression the compound may be removed from around the tooth and dropped into its proper place in the impression. The paper gives in detail the method of making the wire cribs and springs, illustrating with drawing the different processes. For a complete description the reader is referred to the transactions of the association, as it will be published in full there with the necessary drawings.

The use of copper binding wire in making these cribs has convinced the author that copper has a decided tendency to reduce sensitiveness of the teeth where an appliance comes in contact with parts of the teeth unprotected by enamel; and it is suggested that if copper amalgam put into rubber plates where the plate comes in contact with sensitive teeth will have a desirable effect.

The advantages Dr. Jackson claims for the "crib" system, are, young patients can wear it with less inconvenience than any other form of appliance; additional retaining devices and springs can be readily added at any time. It is easily retained and does not interfere with the articulation.

Two other papers were reported to be read under this section, but for lack of time they were withdrawn by the authors, much to the regret of the officers of the section as they were both valuable and interesting.

For lack of time there was no discussion of any of the papers of this section.

Dr. Frank Abbott in lieu of a report from Section IV, Histology and Microscopy, read a paper by Carl Heitzman and Frank Abbott on

SENILE ATROPY OF THE UPPER JAW.

In advanced age the human organization is reduced in size and weight; the whole body as well as its individual tissues shrivels. The question is where does this atropy take place? and what are its visible signs under the microscope? The upper jaw of a woman, who died at the age of 75 years, was placed in a one-half of one per cent. solution of chromic acid for several weeks to soften the bony parts. Vertical sections gave striking views of advanced senile atropy. The medullary spaces varied in size and were filled with either a delicate fibrous connective tissue, or a granular matter, probably disintegrated protoplasm. The most striking feature was the presence of portions of hyaline cartilage at the same height as the former bony structure, indicating that it had been produced from it, and was, in a measure, replacing it. Hyaline cartilage is present in the lower jaw in embryonal life, but it is supposed the upper jaw is derived from fibrous connective tissue. Hence it seems remarkable to find hyaline cartilage in old age. This is another proof that in old age there is an inclanation of the tissues to return to the childhood or even intrauterine conditions. Sections of the case referred to show a thinning of the epithelial layer, due to the reduction in the size of the cells; possibly some of the cells were worn away by mastication and never reproduced.

The dense fibrous connective tissue is changed into a faintly granular protoplasmic mass, in which living matter is scanty and pale, apparently on account of a hydropic infiltration. This will explain the flabbiness of the tissues in old age and the gradual loss of living matter. Particles of living matter imbibing serum become detached, disintegrate, and are taken into the blood and lymph circulation and carried away, and in this way the shrinkage of the body takes place, as new matter is not built up as rapidly as the disintegrating process takes place. The blood vessels in old age are very scant. The arteries disappear by a process of thickening and granulation of the different coats which plug the lumen, and the whole is transformed into a solid tract of fibrous connective tissue, and as such is subject to the same process of disintegration as other connective tissue as indicated above. It was not possible to trace the process of the disappearance of the veins in the same way, but it probably occurs much the same. The nerves also undergo the same disintegrating process, but no study could be made upon the axis cylinder of the medulated nerves in this region.

Hyaline cartilage is doubtless a transient or provisional tissue in early life and the same seems to be the case in declining age, since it appears as a mere intermediate tissue between previous bone and ultimate fibrous connective tissue. The bone is absorbed on its periphery by the periosteum, by the formation of new haversian canals in the solid bone and the enlargement of the former haversian system. The borders become corroded and filled with bay-like excavations, which contain protoplasmic bodies of various sizes and shapes. Nowhere in the specimen were the multinuclear giant cells of Kœliker or osteoblasts, seen. The origin of the medullary corpuscles is from previous bone corpuscles which have been deprived of their surrounding bone substance. The medullary corpuscles elongate and become fibrous basis substance, with a simultaneous new formation of blood vessels. This new formation is again broken down and carried away as above. On this theory the interstitial growth of bone finds a rational and satisfactory explanation. All tissues arise from protoplasm of its medullary corpuscle, and all tissues return to this embryonal or medullary condition before they are absorbed. Origin and decline show a striking similarity, and that which is between " three score years and ten," is a struggle, and change of the whole person as well as all his constituent parts.

DISCUSSION.

Dr. Hunt: This is a valuable and satisfactory paper, as it presents to us a rational and harmonious theory, not only for the building of the body, but for its destruction; not only the soft, but the hard structures as well.

Dr. Ingersol: If Dr. Abbott wishes to teach us to grow old gracefully, he certainly has presented us a very attractive theory as to how it is done. We have often wondered what the process of absorption really was. It seems that construction and destruction must be preceded by solution. The temporary teeth afford an excellent illustration. The nature of mature tissue is not the same as embryonic, and I would like to know what takes place in this tissue. Do the same processes in modification take place in these structures? I would like to ask Dr. Abbott why the bones of aged people are so brittle if there is a return to embryonic conditions?

Dr. Abbott: In old age there are fewer cells and the bone corpuscles are infiltrated with lime salts. In the transition of adult to aged bone the process is so gradual, and even in the breaking down at the most active period, there is no actual return to the embryonal condition, but the tendency is always that way and the process is similar.

The next paper was reported under Section V, Materia Medica and Therapeutics, entitled,

ELECTRICITY AS A THERAPEUTICAL AGENT IN THE TREATMENT OF HYPERÆMIA AND CONGESTION OF THE PULP AND PERIDENTAL MEMBRANE.

by Dr. John S. Marshall.

These diseases are common in dental practice and difficult to control by the ordinary means at our disposal when caused by caries, injury, mechanical irritation, etc., and much more difficult when caused by constitutional derangements.

Electricity can be used successfully in the first mentioned cases to allay the inflammation, and in the others to prevent suppuration or the formation of new growths. The galvanic current is specially applicable to these cases. The negative and positive currents of electricity can be used to stimulate resorption of inflammatory products and new growths. In my own mouth a near exposure of the pulp was capped and covered with an oxyphosphate filling; congestion of the pulp and toothache ensued, which was relieved permanently by two one-half hour applications of electricity on succeeding days. The positive pole of the continuous galvanic current was applied to the tooth and the negative pole to the carotid triangle. The current was applied gradually and the pain gradually disappeared. Three years have elapsed and the tooth has given no trouble since and there is no indication of any peculiar sensibility. In one case of congested pulp in a patient of delicate organization the treatment was used successfully. Pericementitis not caused by septic poisoning from a devitalized pulp, in many cases, may be treated by this method with very gratifying results. The treatment is also useful in hyperæmic odontalgia accompanying pregnancy. When used in these cases it should be accompanied with rest in the recumbent position and anodynes.

From three-fourths to one and one-half miliamperes are sufficient in the cases mentioned above, the frequency of the application depending upon the severity of the local symptoms and the susceptibility of the patient, and one treatment every twenty-four hours of from 15 to 30 minutes is usually all that is necessary. The Faradic current is invaluable for determining the vitality of the pulp. It is superior to the mouth lamp. Hypersensitive conditions of the pulp may be diagnosed and isolated certainly and expeditiously. The miliampere meter should always be used to measure the strength of the current. The battery, miliampere meter and electrodes suitable for this treatment may be secured from the McIntosh Battery and Optical Co., of Chicago.

Dr. Corydon Palmer was given the floor to make a request of the association. He said he was grieved and shocked continually at the lavish display of gold in the teeth of people, and he thought it was a disgrace to the dental profession that no more effort was made to prevent this exhibition, and he hoped the members of this association would strive to overcome this objectionable practice.

Another matter which demands our attention is the premature loss of the deciduous teeth. These teeth, even the roots should be kept in place until the permanent teeth are ready to emerge from the gum and supplant them, and he hoped the dental profession would take more interest in this matter and that the wholesale slaughter of these teeth would be stopped.

### CLINICS.

Dr. C. A. Timme, of New York, gave a clinic illustrating his method of making a glass inlay. A low fusing porcelain is fused into a matrix made with pure gold, No. 60 foil, by burnishing it with a semi-hard rubber point into a cavity previously prepared with definite borders and no undercuts. The powdered porcelain is then mixed with distilled water so that it will readily flow into all parts of the matrix, and is placed in the matrix with a small pointed brush. The matrix with the porcelain in place is then placed on a little platinum disk which is perforated to allow the flame to pass through it; the platinum serves to prevent fusing the gold foil, and the whole held with a pair of tweezers in the flame of a bunsen burner or lamp until the porcelain fuses and fills the matrix; two burnings are usually necessary as the first shrinks considerably. The matrix is then allowed to cool and the gold foil stripped off and the glass filling is ready for insertion in the tooth. Slight undercuts are then made in the cavity and enough thin cement used to fasten the inlay in place. There will usually be sufficient roughness on the underside of the inlay to furnish attachment for the cement. A variety of shades are supplied with the material so that any required color may be readily obtained. This promises to be a ready and successful method of making these inlays.

Dr. Timme also uses this material for making faces for all gold crowns; this he does by cutting out the face of the crown and soldering in a piece of platinum, which makes a shallow cavity, and into this the porcelain is fused, much as the porcelain onto a watch dial. The material cannot be used on less than twenty carat gold.

Dr. V. H. Jackson gave a clinic on making the cribs and springs used in regulating teeth. An intelligible description is not possible without drawings to illustrate. The apparatus and tools necessary are to be found in almost every laboratory. Plate shears, pliers, soldering-iron, block tin or soft solder, taggers' tin, and piano wire, muriate of zinc. The joints are all made by lapping the wires and wrapping them with narrow strips of taggers' tin, and saturating this joint with the muriate of zinc, applying a good sized piece of solder and melting it at once with the hot soldering-iron. This is all done while the fixture is in place on the plaster cast. Care must be observed to not apply the muriate of zinc until ready to apply the hot soldering-iron as it corrodes the piano wire very quickly and prevents a successful job of soldering. It is a very quick and easy way of making these appliances, and extra springs or retaining appliances can be quickly added at any time.

Dr. Truman W. Brophy treated a case of disease of the antrum by perforating it through the alveolar process with a trephine. The treatment suggested was the usual detergents, disinfectant and curative remedies. The case had been previously treated, but had not been properly trephined to secure free drainage.

DR. E. A. STEBBINS, of Massachusetts, exhibited a patient, a boy, in whose mouth he had treated all incipient caries of both the deciduous and permanent teeth with nitrate of silver, and had successfully prevented the continuation of the decay. The method he has used is to apply to the cavity after removing a portion of the softened material powdered nitrate of silver in the presence of the ordinary moisture of the tooth, or, if the rubber is applied a drop of water is added to dissolve the nitrate of silver and allow it to combine with the tooth structure forming the black eschar which has been so successful in preventing further progress of decay. The Doctor has been practicing this method for three years, and he exhibited a great many teeth in which the cavities he has treated showed no signs of returning caries, while other teeth in the same mouth or even the same tooth in other places not treated, was largely decayed. These teeth were all deciduous teeth that he had knowledge of and had watched until they were extracted to make space for the permanent teeth.

DR. ROLLO KNAPP, of New Orleans, exhibited some fine specimens of bridge work, a convenient case for holding instruments and materials for the bridge worker, a new form of bunsen burner, capable of producing any form of flame or any quantity from a small jet to a powerful heat, it is really a combination of several small bunsen burners attached to a plate in such a way that one or all may be used, a broad or concentrated flame may be had at pleasure. He also exhibited a neat device for washing all blood from the fixtures in a fountain spittoon, and controlling by a valve which is worked by pressing an electric button on the arm of the operating chair the water supply.

The meeting was a large and successful one, but it is much to be regretted that so much time had to be consumed in mere business details and discussions of them and that more was not allowed to the discussions and reading of scientific papers. Many valuable papers were offered and not read, and there was no time allowed for discussion of many that were read. It is to be hoped that the new amendments offered this year, to the constitution and by-laws, will remedy this serious defect.

The association elected Dr. W. W. Walker, of New York City, President, and will meet next year at Niagara Falls.

